# Nutrient dynamics in coral symbiosis depend on both the relative and absolute abundance of Symbiodiniaceae species

**DOI:** 10.1186/s40168-022-01382-0

**Published:** 2022-11-07

**Authors:** Shelby E. McIlroy, Casey P. terHorst, Mark Teece, Mary Alice Coffroth

**Affiliations:** 1 School of Biological Sciences, Swire Institute of Marine Science, The University of Hong Kong, Hong Kong SAR, China; 2 Graduate Program in Evolution, Ecology and Behaviour, University at Buffalo, Buffalo, NY 14260 USA; 3 Department of Biology, California State University, Northridge, CA 91330 USA; 4 Department of Chemistry, State University of New York College of Environmental Science and Forestry, Syracuse, NY 13210 USA; 5 Department of Geology University at Buffalo, Buffalo, NY 14260 USA

**Keywords:** Symbiosis ecology, Coral, Nitrogen, Nutrient exchange, Mutualism, Stable isotopes

## Abstract

**Background:**

Symbionts provide a variety of reproductive, nutritional, and defensive resources to their hosts, but those resources can vary depending on symbiont community composition. As genetic techniques open our eyes to the breadth of symbiont diversity within myriad microbiomes, symbiosis research has begun to consider what ecological mechanisms affect the identity and relative abundance of symbiont species and how this community structure impacts resource exchange among partners. Here, we manipulated the *in hospite* density and relative ratio of two species of coral endosymbionts (*Symbiodinium microadriaticum* and *Breviolum minutum*) and used stable isotope enrichment to trace nutrient exchange with the host, *Briareum asbestinum*.

**Results:**

The patterns of uptake and translocation of carbon and nitrogen varied with both density and ratio of symbionts. Once a density threshold was reached, carbon acquisition decreased with increasing proportions of *S. microadriaticum*. In hosts dominated by *B. minutum*, nitrogen uptake was density independent and intermediate. Conversely, for those corals dominated by *S. microadriaticum*, nitrogen uptake decreased as densities increased, and as a result, these hosts had the overall highest (at low density) and lowest (at high density) nitrogen enrichment.

**Conclusions:**

Our findings show that the uptake and sharing of nutrients was strongly dependent on both the density of symbionts within the host, as well as which symbiont species was dominant. Together, these complex interactive effects suggest that host regulation and the repression of *in hospite* symbiont competition can ultimately lead to a more productive mutualism.

Video Abstract

**Supplementary Information:**

The online version contains supplementary material available at 10.1186/s40168-022-01382-0.

## Background

Symbiosis is a source of evolutionary innovation in which an entire ecological/biological repertoire can be acquired through close association with other species. These partnerships yield trait combinations that can expand access to nutrients, provide protection from enemies, and/or aid in mobility and dispersal. In corals, endosymbiotic dinoflagellate algae of the family Symbiodiniaceae supply the majority of the host’s energetic needs via photosynthesis. Symbiodiniaceae benefit from access to a light-rich habitat and inorganic nitrogen sources in the form of the host’s metabolic waste. This combination of trophic strategies (i.e., algal photosynthesis and coral heterotrophy) and efficient recycling of nutrients between the partners underpins the success of corals in nutrient-limited tropical oceans. However, anthropogenic stressors have exposed the vulnerability of this critical symbiosis to environmental change resulting in global coral bleaching and mortality events [1, 2], but flexibility within this symbiosis may prove to be its salvation [3].

The identity and relative abundance of algal symbionts define many aspects of coral physiology, including growth [4–6], disease [7], and bleaching [8]. Comparative studies of corals and other cnidarians hosting one Symbiodiniaceae species or another have demonstrated differences among symbiont species in their contributions to host metabolism, with variations in both the amount of carbon [9–12] and assimilated inorganic nitrogen [11] transferred to the coral. As genetic techniques open our eyes to the breadth of symbiont diversity within myriad microbiomes, our understanding of the ecological mechanisms that restrict and maintain diversity in symbiosis has not kept pace [13]. Most corals maintain some capacity for symbiont flexibility [14], and diversity of symbionts at background abundances is common [15]. Yet it remains poorly understood how ecological interactions among co-occurring symbionts, and between each symbiont and their host, will influence the balance of costs and benefits of the association.

Natural selection on symbionts alone is not expected to select for beneficial partners, instead, as in all ecological systems, selection should favor symbionts best able to exploit their host and outcompete other symbionts [16]. As such, unregulated symbiont diversity not only exposes the host to sub-optimal symbionts but also to the consequences of antagonism among symbionts competing for space within the host [17]. Recent work demonstrated that symbionts alter their metabolism in vast ways when a competing symbiont species is introduced in culture, shifting investment of newly assimilated nutrients between compounds that support growth vs. storage [18]. If those competitive behaviors persist for symbionts within coral tissues, they may negatively affect the quality and persistence of the symbiosis. The result of competition may prevent the coral from associating with the most beneficial symbionts, divert nutrients from the coral-algal exchange, and/or potentially expose the host to allelopathic compounds. Although multiple symbiont cells can reside within a single host cell, individual symbionts are bound by a multilayered symbiosome membrane that serves as the bridge for transport of nutrients, gases, and photosynthetically fixed carbon. This membrane helps to maintain metabolic stability within symbiosis [19] and may also inhibit the ability of symbionts to detect the presence of potential competitors.

It is critical for the stability of the mutualism that corals and other hosts develop mechanisms to both avoid conflict among symbionts and to avoid associations with less beneficial symbionts. To ensure the highest return on their investment, many hosts have evolved mechanisms to allocate more resources to highly cooperative symbionts and/or to inhibit associations with more parasitic symbionts. These host sanctions and rewards are two mechanisms by which symbiotic interactions can be stabilized on ecological timescales [20]. For example, soybeans can reduce growth of intracellular rhizobia that fail to fix nitrogen [21], while other legumes can reward specific fungal partners with more carbohydrates [22]. Corals have some capacity to regulate nutrient availability to their symbionts to inhibit over-proliferation of less beneficial symbionts *in hospite* [23, 24] and ensure the release of sufficient photosynthetic carbon to the host [25]. But the ability of corals to use these mechanisms to regulate community composition remains unknown.

In most corals, symbiosis is initiated via exogeneous uptake of symbionts in the early stages of ontogeny. Over time, what can initially be a diverse community winnows towards a relatively predictable, stable association [26, 27]. The octocoral *Briareum asbestinum* provides a well-studied model for symbiont dynamics in an obligate mutualism, in part because of its ability to associate with several symbiont species, including but not limited to *Symbiodinium microadriaticum* and *Breviolum minutum*. Furthermore, *B. asbestinum* juveniles have documented patterns of symbiont uptake and succession in both the field and the laboratory [26, 28], with outcomes that indicate that host control [29], symbiont competition [30], and environmental context [30] play a role in shaping the symbiont community. Here, we use stable isotope tracers of carbon and nitrogen to track the nutritional currency exchange between host and symbionts as a function of symbiont community diversity to examine how nutrient sharing in the symbiosis is affected by symbiont diversity and better understand the ecological processes that shape this symbiosis.

## Methods

*Briareum asbestinum* is a common Caribbean octocoral that releases aposymbiotic larvae that acquire symbionts from the environment in the following weeks-years [26]. In May of 2013, larvae were collected from the surfaces of approximately 30 *B. asbestinum* colonies on a shallow reef (~2 m depth) along Long Key, Florida, and transported back to the Keys Marine Laboratories (KML; Long Key, FL, USA). Once in the laboratory, larvae were rinsed and maintained for the duration of the experiment in artificial seawater (ASW; Instant Ocean®; salinity: 36 ppt) to prohibit exposure to environmental sources of Symbiodiniaceae. For the first 48 h, larvae were maintained in plastic containers with noncirculating but regularly refreshed ASW. The larvae were transferred to 1.5-L sterile, plastic water bottles (~300 larvae L^−1^) and transported to the University of Buffalo culture facilities where they settled onto cleaned gorgonian axial branches, a common natural settlement substrate, which had been collected in the field and sterilized. Once larvae had settled and metamorphosed into polyps (~2 weeks following collection), hereafter referred to as “recruits,” the branches were distributed among 1-L containers with 600 mL ASW (*n* = 32 containers with 30–40 *B. asbestinum* recruits each).

### Symbiodiniaceae inoculations

Two cultured Symbiodiniaceae species were selected from the BURR Culture Collection at the University at Buffalo: *Symbiodinium microadriaticum* (Culture ID: 04-503; cpType: A194, [31]; ITStype: A1 [32];) and *Breviolum minutum* (Culture ID: Mf01.05b; cpType: B184; ITStype: B1). Each of these species has the ability to establish sustained symbioses with *B. asbestinum* in previous laboratory studies [28, 30]. They have also been found in naturally established *B. asbestinum* juveniles in the field but are generally displaced by other Symbiodiniaceae species within months following settlement [26, 33]. However, those homologous symbionts have yet to be successfully brought into culture.

Previous labwork demonstrated that *S. microadriaticum* is displaced from the symbiosis with *B. asbestinum* following exposure to *Breviolum* species including *B. minutum* [28, 30]. Therefore, we varied the timing of initial exposure to *B. minutum* (months to weeks before the isotope tracer experiment) as well as the relative ratio of symbiont species inocula (*S. microadriaticum*:*B. minutum* target ratios — 100:0, 90:10, 75:25, 50:50, 25:75, 10:90, 0:100) to generate recruits with an array of single and mixed symbiont species associations. The actual abundance and ratio of symbiont species were determined for each individual recruit using qPCR (see below). For 3 months, recruits were maintained in artificial seawater (ASW; Instant Ocean®, Blacksburg, VA, USA) at 28 °C, beneath four fluorescent T5 grow lights (measured at 165–190 photons m^−2^ s^−1^ with a LI-COR optical sensor) on a 14/10 h light:dark cycle. Throughout this time, regular (2–3 times per week) maintenance included full ASW changes and symbiont inoculations at total densities of 200 cells/ml. Recruits were also fed weekly with Zeigler® Larval AP100 Diet (Zeigler Bros., Inc.) during the first 2 months of maintenance, but feeding was discontinued 1 month prior to the isotope tracer experiment.

### Pulse chase with dual isotope tracers

**Fig. 1:**
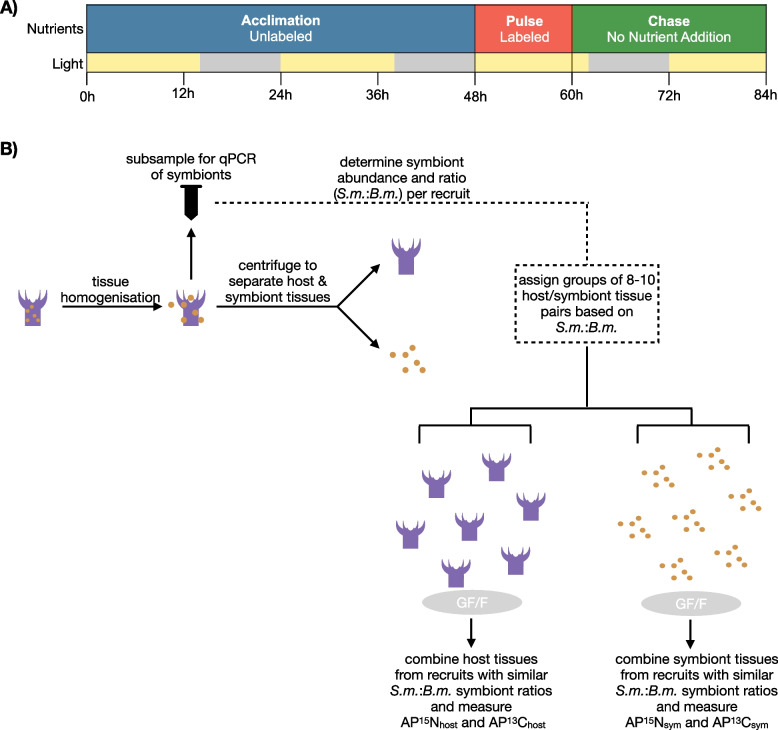
Experimental and sampling design. **A** Nutrient and light cycling conditions (yellow—on, gray—off) throughout the hours of the pulse-chase experiment. Samples were processed at 84 h. **B** Sample processing schematic for qPCR and subsequent stable isotope analyses. Host and symbiont tissues for each recruit were separated, and a subsample was used for quantitative analysis of each symbiont type. Using the relative ratio of symbiont types, groups 8–10 recruits were assigned, and host or symbiont tissues were combined into a glass fiber filter (GF/F)

A pulse-chase experimental design was used to trace the assimilation and transfer of nitrate (^15^NO_3_) and bicarbonate (H^13^CO_3_) in *B. asbestinum* recruits (Fig. 1A). First, *B. asbestinum* were acclimated for 2 days in ASW supplemented with non-isotopically enriched nitrate and bicarbonate to stimulate the induction of nitrate-reducing enzymes [34]. They were then transferred to isotopically enriched ASW for a period of 12 h of light (pulse phase). Finally, they were rinsed under flowing ASW to remove unincorporated compounds from their surfaces and transferred to ASW for 24 h (chase period). Pilot studies were used to determine the appropriate duration of the pulse and chase period and to determine the amount of tissue necessary for accurate nutrient readings.

Each experimental chamber consisted of a 1-l glass beaker with 0.7 L of stirring seawater. Within this, we added branch(es) with 30–40 *B. asbestinum* recruits. A 17-l stock solution of media for each stage of the experiment (acclimation, pulse, chase) was prepared at the start and used for all replicates. Nutrient supplementation at [NO_3_^−^] = 10 μM and [HCO_3_^−^] = 0.4 mM was included in the acclimation media, using non-isotopically enriched compounds, and in the pulse media using isotopically enriched compounds. No supplemental nutrients were added in chase media (Table S1). Laboratory lighting was maintained at (165–190 photons m^−2^ s^−1^; Licor) on a 14:10 h light:dark cycle. Four chambers were run in parallel, and the entire acclimation/pulse/chase routine was repeated five times over 2 weeks for a total of 20 experimental replicates. As a control, we included a set of dark incubations which were covered by a black-out box for the entirety of the pulse period to inhibit photosynthesis and confirm the symbiont as primary source of traced nutrient incorporation (Fig. S1). Water samples were taken from each of the stocks throughout the experiment for analysis of NO_3_-N, NH_3_-N, TDN, and DIC at the Penn State Institutes of Energy and the Environment (Table S1).

At the end of the experiment, *B. asbestinum* were removed at their base from branches, rinsed twice in 0.2-μm filtered DI water, transferred into 600 μl of 0.2-μm filtered DI water, and macerated with a Tissue Master 125 (Omni International). One-hundred microliters of homogenized tissues was subsampled, preserved in 90% EtOH, and stored at −20 °C for subsequent genetic analysis. The remaining homogenate was separated into host and symbiont fractions by a series of centrifugation and washing steps (Fig. 1B). First symbionts were pelleted at 1000 rcf, and the supernatant with host tissue was removed and frozen. The pellet was then rinsed twice in DI water and frozen. A total of 630 *B. asbestinum* recruits were processed.

### Quantitative PCR

Genomic DNA was extracted from homogenate tissue subsamples using a CTAB protocol optimized for Symbiodiniaceae extraction [35]. Quantitative PCR (qPCR) using genera-specific primers and probe sets were used to quantify the total copies of each of the *Symbiodinium* and *Breviolum* actin genes [36]. To convert actin gene abundance to absolute cell numbers, and account for interspecies differences in actin copy number, standards made from genomic DNA extracted from cultured *S. microadriaticum* and *B. minutum* were also prepared from 2000, 8000, 16,000, 64,000, 19,2000, and 384,000 cells.

Reaction volumes were 10 μl with 5 μl TaqMan Genotyping Master Mix and 1 μl genomic DNA template. Assays were optimized for each target including the following: *Symbiodinium* — 150 nM forward primer, 100 nM reverse primer, and 150 nM probe and *Breviolum* — 200 nM forward primer, 300 nM of reverse primer, and 100 nM probe. All qPCR reactions were performed using a CFX Real-Time PCR Machine (Biorad) with 384-well module with an initial incubation (2 min at 50 °C, 10 min at 90 °C) followed by 40 cycles of 10 s at 95 °C and 1 min at 60 °C. Cycle threshold (C_T_) values for each assay were calculated using an automatic baseline interval and relative fluorescence threshold of 0.01. Each sample was run in duplicate, and each plate included a full set of replicated standards for each symbiont species. Spearman’s rank correlation was used to test for a correlation between symbiont density (the number of cells per recruit) and symbiont ratio (the relative ratio of *S. microadriaticum* to *B. minutum*).

### Stable isotope analysis (SIA)

We determined that 8–10 *B. asbestinum* recruits were required to obtain enough host and symbiont biomass for accurate determination of C and N isotopic ratios. We used the symbiont ratio as a basis to combine 8–10 paired host or symbiont tissues from individual recruits onto a filter for SIA analyses (Fig. 1B). Samples were prepared for SIA by filtering onto precombusted 0.7 μm glass fiber filters (Whatman GF/F), rinsed once with 1% HCl to remove inorganic carbonate from residual media, rinsed three times with DI water, and then folded, wrapped in foil, frozen, and transferred to SUNY-ESF.

At the Environmental Science Stable Isotope Laboratory (EaSSIL) at SUNY-ESF, samples were lyophilized (−60 °C, 48 h, 150 torr) and packed into tin capsules. Using a Costech elemental analyzer linked via a ThermoFinnigan ConFlo III interface to a Finnigan MAT Delta XL Plus stable isotope mass spectrometer (EA-IRMS), we determined total organic carbon content, total organic nitrogen content, δ^13^C, and δ^15^N values of host, symbionts, and nutrient solutions. The precision of the stable isotope measurements (expressed in the standard per mil notation) was verified using the National Institutes of Standards and Technology RM8573 (δ^13^C = −26.4 ± 0.1‰, δ^15^N = −4.5 ± 0.3‰, *n* = 38), and RM8574 (δ^13^C = +37.3 ± 0.3‰, δ^15^N = +47.6 ± 0.3‰, *n* = 38). Daily precision of the instrument was verified by repeated analyses of internal laboratory standards including acetanilide (δ^13^C = −33.4 ± 0.2‰, δ^15^N = −1.0 ± 0.1‰, *n* = 32), valine (δ^13^C = −10.7 ± 0.2‰, δ^15^N = −6.6 ± 0.1‰, *n* = 9), and daphnia (δ^13^C = −24.4 ± 0.2‰, δ^15^N = +17.4 ± 0.3‰, *n* = 8), during the sample runs.

### Data analysis

Enriched values are reported as atom percent of the heavy isotope (AP^15^N & AP^13^C), which is calculated by the count of the heavy isotope relative to the total number of atoms of N or C in the sample. We also calculated the total milligrams of newly assimilated nitrogen per recruit (N_new_), by multiplying AP^15^N (per mille) by the total organic nitrogen (mg) for each of the tissue fractions and summing them with the assumption that N is highly retained in the symbiosis through the duration of the experiment, whereas carbon may be respired over time [37].

$$Total\ {N}_{new}/ recruit=\frac{\left( total\ organic\ {N}_{host}(mg)\times \frac{\delta^{15}{N}_{host}}{1000}\right)+\left( total\ organic\ {N}_{sym}(mg)\times \frac{\delta^{15}{N}_{sym}}{1000}\right)}{Total\# recruits/ filter}$$

Multiple linear regression was used to test if symbiont density (number of symbionts per recruit), symbiont community composition (symbiont ratio = the relative ratio of *S. microadriaticum* and *B. minutum* cells [*S. m.*:*B. m.*] determined by qPCR), or their interaction were significant predictors of the response variables AP^13^C_host_, AP^13^C_sym_ AP^15^N_host_, AP^15^N_sym_, and Total N_new/recruit_. When a significant interaction term was identified, we used the interflex package in R (Hainmuller et al. 2021) to generate marginal effect plots with 95% confidence intervals and visualize how the effect of one predictor variable changed with each level of the other predictor variable. Using the confidence intervals, we approximated where the slope was significantly different from 0, partitioned the data accordingly, and plotted the data with the significant and nonsignificant linear relationships of partitioned groups. All data were confirmed to meet the assumptions of homogeneity of variance and linearity, with the residuals approximately normally distributed.

## Results

### Symbiont density and relative abundance

**Fig. 2:**
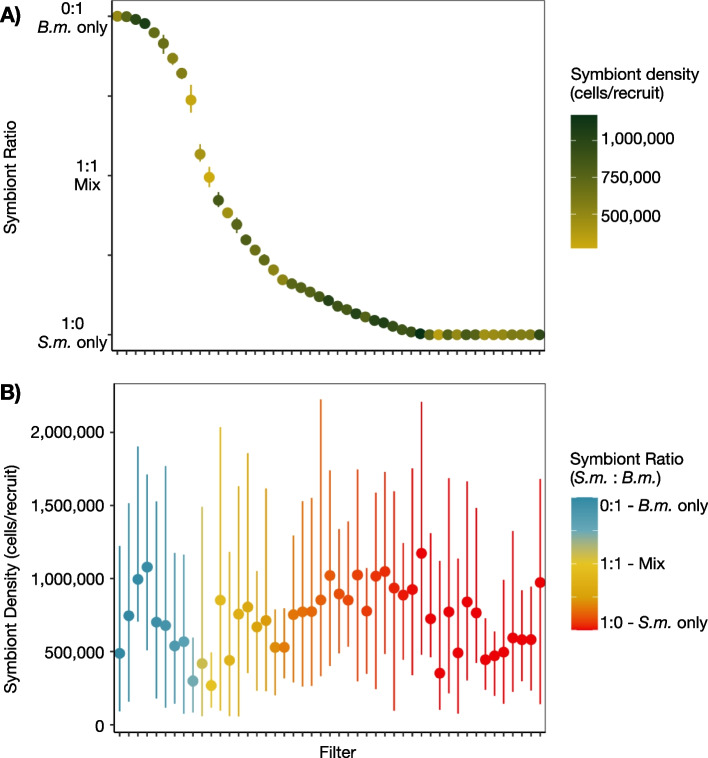
Recruit combinations for SIA. To obtain enough mass, separated host or symbiont tissues from 8 to 10 recruits were combined onto a glass fiber filter prior to stable isotope analysis. Variation in the **A** relative ratio of symbionts (*S. microadriaticum*:*B. minutum)* and **B** symbiont density (cells/recruit) for the combined recruits is shown for each filter along the x-axis. The mean (points) and range (bars) of data are shown. Combinations were based on similarity in the relative ratio of symbionts as measured by qPCR; host and symbiont tissue combinations were maintained to generate host and symbiont filter pairs

Single-strain inoculations yielded ~50 recruits each hosting either *S. microadriaticum* or *B. minutum*. Of these, 20 recruits with only *S. microadriaticum* and 20 with only *B. minutum* were used as dark controls and the rest in the main experiment. The regime of varying the timing of exposure and ratio of *S. microadriaticum* and *B. minutum* used for mixed inoculations was successful in establishing ~350 recruits of mixed symbiont species assemblages at various symbiont ratios (*S. m.:B. m.*) (Fig. 2A). However, an artifact of delaying exposure to *B. minutum* was an overrepresentation of recruits with higher ratios of *S. microadriaticum*, including an additional 25 recruits in which only *S. microadriaticum* was detected. This occurred most often in treatments in which *B. minutum* exposure occurred only in the final weeks preceding the pulse-chase experiment. Mean symbiont densities across all recruits ranged from 268,738 to 1,387,584 cells/recruit, and the density of symbionts was not correlated with symbiont ratio (Spearman’s rank correlation rho_(584)_ = 0.01, *p* = 0.83; Fig. 2B). Therefore, we grouped 8–10 recruits onto filters based on their similarity in *S. m.:B. m*. This resulted in 59 pairs of host tissue and symbiont tissue filters on which we performed stable isotope analysis. The maximum range of *S. m.:B. m.* values among individual recruits combined onto a single filter was 4.7% with a mean range of *S. m.:B. m.* of 2% (Fig. 2A).

### AP^13^C enrichment

**Fig. 3:**
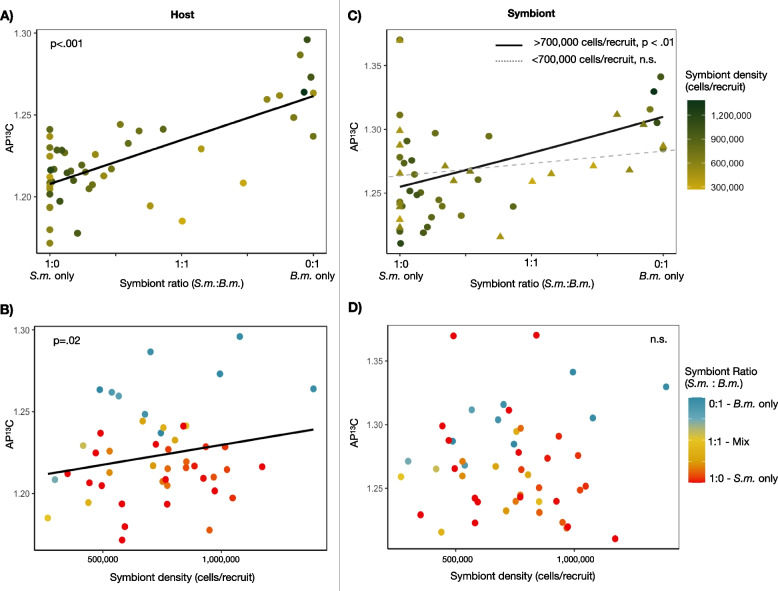
Atom percent ^13^C values for host (left) and symbiont tissues (right) based on symbiont ratios (the relative ratio of *S. microadriaticum* and *B. minutum* symbionts, top) and symbiont densities (total cells per recruit, bottom). Solid black lines show significant relationships as determined by generalized linear models, with *p*-values reported in each plot. In the case of an interactive effect, the primary response is divided into levels of the secondary response variable and then modeled and plotted independently for levels of nonsignificant (triangles and dashed lines) and significant (circles and solid line) effects

A high proportion and abundance of *B. minutum* lead to higher carbon assimilation rates in both host and symbiont tissues. In host tissues, both an increasing proportion of *B. minutum* (*p* < 0.001) and, to a lesser extent, an increasing density of symbionts overall (*p* = 0.021) were positively correlated with AP^13^C_host_ (F(3.44) = 28.6, *p* < 0.001) with the model explaining 54% of the variance (Fig. 3A and B). However, in symbiont tissues, there was a significant interaction between symbiont ratio and symbiont density on AP^13^C_sym_ (*p* = 0.035). In this case, the model explained 17% of the variance (F(3.44) = 4.26, *p* < 0.01) increasing to 37% of the variance explained where two identified outliers were removed (F(3.42) = 9.86, *p* < 0.01). Marginal effect plots indicated that as *B. minutum* increased in proportion, so did carbon AP^13^C_sym_, but that this only occurred when symbiont densities were higher than ~700,000 cells per recruit (Fig. 3C and D, Fig. S2).

### AP^15^N enrichment

**Fig. 4:**
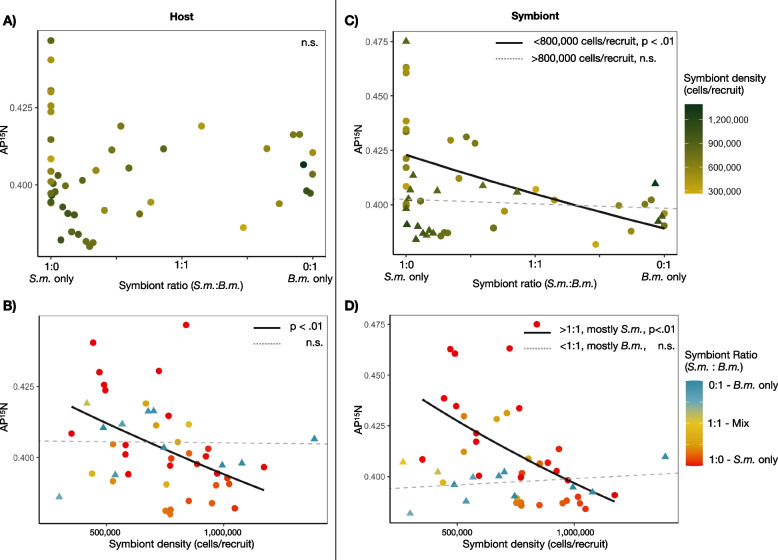
Atom percent ^15^N values for host (left) and symbiont tissues (right) based on symbiont ratios (the relative ratio of *S. m.* and *B. m.* symbionts, top) and symbiont densities (total cells per recruit, bottom). Solid black lines show significant relationships as determined by generalized linear models, with *p*-values reported in each plot. In the case of an interactive effect, the primary response is divided into levels of the secondary response variable and then modeled and plotted independently for levels of nonsignificant (triangles and dashed lines) and significant (circles and solid line) effects

Symbiont ratio and density were shown to be interactive in predicting the amount of newly assimilated nitrogen in both host and symbiont tissues. In host tissues, the full interactive model significantly (F(3.43) = 3.25, *p* = 0.03) explained 17% of the variance in measured AP^15^N_host_ with a significant effect of symbiont density (*p* < 0.01) and a significant interaction term (*p* = 0.04; Fig. 4A and B). Marginal effect plots indicate that the overall trend in nitrogen enrichment varied depending on the dominant symbiont species (Fig. S3). In recruits with > 50% *B. minutum*, AP^15^N_host_ did not scale with symbiont densities. However, in recruits with > 50% *S. microadriaticum*, increasing densities of symbionts per host led to higher values of AP^15^N_host_.

For symbiont tissues, significant model terms included symbiont density (*p* < 0.001) symbiont ratio (*p* < 0.001) and their interaction (*p* = 0.002) explaining 31% of the variance (F(3.44) = 8.16, *p* < 0.001; Fig. 4C and D). Marginal effect plots indicate that within less densely populated hosts, i.e., those harboring less than ~800,000 symbionts per host, AP^15^N_sym_ was lower in hosts dominated by *B. minutum* and increased with increasing proportions of *S. microadriaticum* (Fig. S4). Concomitantly, within those hosts dominated by *S. microadriaticum*, increasing symbiont densities resulted in significant decreases in AP^15^N_sym_ (Fig. S5).

### Nutrient sharing

**Fig. 5:**
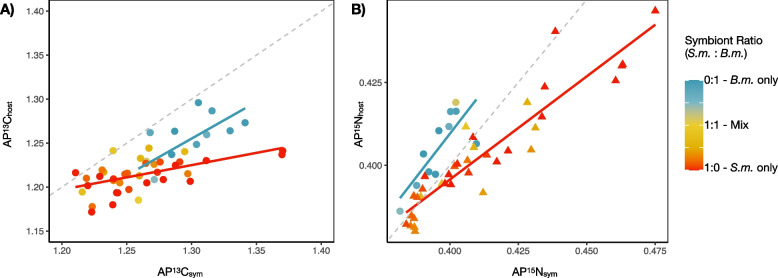
Comparison of host and symbiont isotopic enrichment of **A** ^13^C and **B** ^15^N. Red and blue lines show linear fit to data of recruits dominated by either *S. microadriaticum* or *B. minutum*, respectively. Dotted line shows a 1:1 relationship

We examined the relationship between host and symbiont tissue enrichment by plotting AP^13^C_sym_ by AP^13^C_host_ and modeling that relationship for *S. microadriaticum*-dominated and *B. minutum*-dominated hosts (Fig. 5A). Both showed a positive correlation between symbiont and host enrichment, but the slope of the relationship was higher in *B. minutum*-dominated hosts than those dominated by *S. microadriaticum* (0.83 and 0.28, respectively; Fig. 5A). Similar trends were seen for the relationship between AP^15^N_sym_ and AP^15^N_host_ dominated by either *B. minutum* or *S. microadriaticum* (1.07 and 0.62, respectively; Fig. 5B).

### Total N_new_

**Fig. 6:**
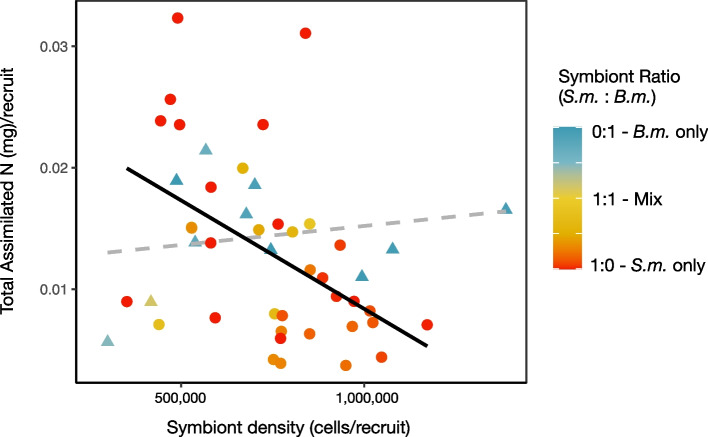
Total amount of newly assimilated nitrogen (mg) from combined host and symbiont tissues. Solid black line shows significant relationship as determined by generalized linear models, with *p*-values. To visualize the significant interaction between symbiont density and symbiont ratio, data for symbiont ratios are plotted independently for levels of nonsignificant, < 1:1, mostly *B. minutum* (triangles and dashed lines), and significant > 1:1, mostly *S. microadriaticum* (circles and solid line) effects

The sum of the newly assimilated nitrogen will significantly be affected by symbiont density (*p* = 0.03) with a significant interaction between symbiont density and ratio (*p* = 0.02); this full interaction model explained 20% of the variance (F(3.41) = 4.61, *p* < 0.01; Fig. 6). Marginal effect plots indicate that in hosts dominated by *S. microadriaticum*, increasing symbiont densities was negatively correlated with Total N_new_ (Fig. S6).

## Discussion

Recent research has highlighted the complex and principal role of nutrient exchange among coral hosts and their algal symbionts in maintaining a functional symbiosis [6, 23, 38]. In this study, we found that nutrients were largely affected by interactive effects of total symbiont density and the relative ratio of symbiont species. For example, only after a symbiont density threshold was achieved was carbon shown to scale with the relative ratio of symbionts, increasing with the relative ratio of *B. minutum*. Conversely, nitrogen dynamics were characterized by the dominant symbiont species. Those hosts dominated by *B. minutum* received consistent nitrogen resources independent of symbiont density, whereas increasing symbiont densities negatively impacted nitrogen assimilation in hosts dominated by *S. microadriaticum*. But rather than evidence for interspecific competition among symbionts, these species-specific patterns in nutrient cycling provide a mechanism by which nutrient dynamics may be involved in host regulation of symbiont community structure.

Recent study of Symbiodiniaceae species in co-culture demonstrated that interspecific competition altered nutrient assimilation rates and subsequent compound production of *Cladocopium goreaui* and *Durusdinium trenchii* [18]. If these interactions are sustained within the host, where nitrogen is limited [39], they have the potential to destabilize the symbiosis through alterations in both nutrient acquisition and sharing. Instead, *in hospite* cohabitation of *S. microadriaticum* and *B. minutum* within the tissues of host *B. asbestinum* did not show a competitive shift in metabolism. After a threshold density of symbionts was reached, the production and sharing of photosynthetically derived carbon were well predicted by the ratio of each symbiont type with mixed communities providing intermediate levels of carbon resources (Fig. 3A and C). An increased number of co-dominated (*S. m.*:*B. m.* ~1:1) recruits may provide a more clear and potentially nonlinear fit to this relationship; however, the observed effect remains far smaller than that expected from co-cultured Symbiodiniaceae experiments (McIlroy et al. 2020). While multiple symbiont cells can exist within a single host cell [40], the symbiosome may serve to physically isolate individual symbionts [41]. The additive relationship observed here suggests that interactions among *in hospite* symbionts are indeed restricted. A similar pattern was seen in radiotracer experiments in the giant sea anemone where significant differences in carbon translocation (^14^C) occurred between anemones harboring “type A” or “type B” Symbiodiniaceae, while mixed symbiont assemblages (A + B) translocated intermediate levels of carbon relative to the two monophyletic groups [9]. Both our study and Loram et al. (2007) assessed isotopic values of bulk tissues of mixed symbiont communities. It is therefore possible that antipodal responses of symbiont species would have gone undetected [18]. Ultimately however, interactions among codominant symbionts, if any, did not interfere with the balance of carbon in the symbiosis more generally. Carbon translocation (AP^13^C_host_) mirrored that of symbiont tissues (AP^13^C_sym_) with carbon enrichment values increasing linearly with increasing proportions of *in hospite B. minutum* to *S. microadriaticum* symbionts (Fig. 3A and C).

We found that *S. microadriaticum* was not only less productive but also more selfish with carbon resources (Fig. 5A), as has been reported in other hosts [42, 43]. In *Briareum asbestinum*, hosts that harbor *B. minutum* have been shown to have higher survival rates than those that harbour other symbiont species [28]. This link between variation in symbiont productivity and host survival provides a basis for natural selection in favour of hosts that can limit long-term associations with *S. microadriaticum* and/or promote associations with *B. minutum*. Indeed, in both the lab and field, *B. asbestinum* transition to *Breviolum*-dominated symbiont communities [26, 28]. This occurs in spite of the fact that *S. microadriaticum* can infect corals sooner and at higher densities than other symbiont species [30]. While other studies have suggested that increasing symbiont densities can cause self-shading, and limit photosynthetically driven carbon assimilation [36], this was not seen in our study. Instead, symbiont densities did not affect AP^13^C_sym_ and led to slight but significant increases in AP^13^C_host_ (Fig. 3). Symbiodiniaceae are capable of maintaining high rates of photosynthesis across light conditions by increasing the efficiency of light harvesting and utilization [44, 45]. Hosts with higher symbiont densities may also represent later stages in the onset of the symbiosis wherein symbiont cells drastically reduce cell replication and are able to generate more photosynthates in excess [6, 36].

Although carbon fixation via photosynthesis is essential to the coral-algal symbiosis, in oligotrophic tropical waters, nitrogen availability often limits reef productivity (reviewed in [46, 47]) and has a primary role in regulating symbiont abundance [38, 39]. In our study, the dominant (i.e., the strain present at > 50% ratio) defined patterns of nitrogen assimilation which affected both AP^15^N_sym_ and AP^15^N_host_. Where *S. microadriaticum* was the dominant strain, increasing cell densities resulted in decreasing AP^15^N_host_ and AP^15^N_sym_ (Fig. 4). This shows that *S. microadriaticum* are sensitive to nutrient dynamics with the ability to ramp up assimilation where nitrogen is in excess, a functional trait that may allow them to proliferate quickly in newly settled recruits [30]. Given a limited pool of nitrogen within the host habitat [39], density-dependent uptake may indicate that competition among symbionts (i.e., intraspecific competition among *S. microadriaticum*) drives nitrogen assimilation rates. However, a closer look at the cumulative amount of newly assimilated nitrogen for an individual holobiont, accumulated across both host and symbiont tissues, shows that total amount of new nitrogen assimilated per recruit drops precipitously with increasing abundances of cells in *S. microadriaticum*-dominated hosts (Fig. 6). This suggests that less nitrogen overall was available to be assimilated as *S. microadriaticum* increased in abundance. Critically, this nitrogen limitation was not evident in hosts dominated by *B. minutum* (Fig. 4B and D) where AP^15^N_sym_ and AP^15^N_host_ remained stable across densities.

The cumulative effect of these limitations on nutrient dynamics of the symbiosis was ecologically complex. While AP^15^N in symbiont tissues was higher or equal in *S. microadriaticum*-dominated communities, relative to *B. minutum*-dominated communities, that functional difference did not consistently benefit the host. Instead, at low densities, *S. microadriaticum* communities were most beneficial to the host in terms of nitrogen (highest AP^15^N_host_), but at higher symbiont densities, hosts dominated by *S. microadriaticum* received less nitrogen than those same densities of *B. minutum*-dominated communities (Fig. 4D). This is not the first evidence of selfishness or parasitism of *Symbiodinium* spp. (see [30, 43, 48]).

For long-lived hosts, sequential partnering with multiple symbiont species over time, including sub-optimal species, may have advantages. Acacia trees, for example, benefit from associations with a sterilizing ant species which increases survivorship in early ontogeny and is later replaced by ants that increase fecundity [49]. These accumulated effects on lifetime fitness, however, were dependent on the timing and efficiency of turnover between different partners. While poorly understood, a more drastic example of symbiont turnover occurs in *B. asbestinum* where, by 4 years of age, juveniles have switched between two species of *Breviolum* [29]; a similar phenomenon occurs in *Acropora* juveniles at 3 or more years old [27]. Initial uptake and winnowing may also be an important part of this sequence. Mortality is extremely high for newly settled coral recruits with rates that decrease as coral size increases [50]. Therefore, recruits may initially benefit from highly infectious, and quickly proliferating symbiont species that are ultimately sub-optimal, but only if they can later be replaced by more optimal symbionts. In this case, *S. microadriaticum* at low densities provided the highest nitrogen benefits to their hosts but became less beneficial relative to *B. minutum* as symbiont densities increased. Symbiont types within *Breviolum* ultimately provide the greatest growth and survivorship benefits to *B. asbestinum* hosts [28]. Ontogenetic flexibility in the regulation of symbiont densities [51] and identity [52] has been demonstrated. While a mechanistic understanding of symbiont turnover and the function of diversity in symbiosis is lacking, our stable isotope tracing experiments have provided a snapshot of nutrient transactions between host and symbionts across an array of symbiont community profiles. A host’s ability to regulate symbiont densities *in hospite* is critical for maintaining a stable symbiosis (Cunning & Baker 2014); here, we suggest that there is a species-specific mechanism for this. Technological advances in stable isotope tracing (e.g., NanoSIMS, compound specific stable isotope analyses) and further combinations with genetic techniques (e.g., qPCR, FISH probing, and flow cytometry sorting [18, 53] can provide further insights into the maintenance and restriction of diversity in symbioses across systems.

## Conclusions

This study provides insight into the complex nutrient dynamics that occur between corals and the Symbiodiniaceae that sustain them. While intraspecific competition seemed to be repressed in the host environment, rates of nitrogen assimilation showed dynamic and species-specific regulation. While theory has long suggested that hosts evolve strategies to stabilize mutualisms, these dynamics have been difficult to track. On the basis of our observations, we suggest that the regulation of endosymbiotic communities by coral hosts can lead to more productive mutualisms. As genetic techniques open our eyes to the breadth of symbiont diversity within myriad microbiomes, our understanding of the ecological mechanisms that restrict and maintain diversity in symbiosis must advance. Methodological approaches which provide a more holistic picture of potential conflict and synergy will continue to reveal critical aspects of the ecological and evolutionary dynamics of symbiosis.

## Supplementary Information

**Additional file 1: Table S1.** Nutrient and isotopic compositions of artificial seawater media used for each stage of the experiment. Concentrations were based on Tanaka et al. (2006). All compounds were manufactured and purchased from Sigma-Aldrich ® (St. Louis, MO, USA). **Fig. S1.** Isotope baseline and dark controls. A set of recruits was tested to provide a baseline isotope value and to confirm that symbionts were the primer drivers of inorganic carbon and nitrogen into the symbiosis. Baseline recruits were sampled prior to the start of the experiment, Dark Enriched samples were exposed to isotopically enriched enriched nutriens but kept in the dark to inhibit photosynthesis; Enriched samples show the cumulative data from the final experiment. **Fig. S2.** The significant interaction effect of symbiont ratio and symbiont density on atom percent 13C values of symbiont tissues was examined by plotting the marginal effect of symbiont ratio (S.m.:B.m.) as moderated by symbiont density (cells/recruit). Green bar indicates the range of symbiont densities in which there is a significant positive effect of symbiont ratio on AP13Csym. Gray area shows the 95% CI. **Fig. S3.** The significant interaction effect of symbiont ratio and symbiont density on atom percent 15N values of host tissues was examined by plotting the marginal effect of symbiont density as moderated by symbiont ratio (S.m.:B.m.). Red bar indicates the range of symbiont ratios in which there is a significant negative effect of symbiont density on AP15Nhost. Gray area shows 95% CI. **Fig. S4.** The significant interaction effect of symbiont ratio and symbiont density on atom percent 15N values of symbiont tissues was examined by plotting the marginal effect of symbiont ratio (S.m.:B.m.) as moderated by symbiont density (cells/recruit). Red bar indicates the range of symbiont densities in which there is a significant negative effect of symbiont ratio on AP15Nsym. Gray area shows the 95% CI. **Fig. S5.** The significant interaction effect of symbiont ratio and symbiont density on atom percent 15N values of symbiont tissues was examined by plotting the marginal effect of symbiont density as moderated by symbiont ratio (S.m.:B.m.). Red bar indicates the range of symbiont ratios in which there is a significant negative effect of symbiont density on AP15Nsym. Gray area shows 95% CI. **Fig. S6.** The significant interaction effect of symbiont ratio and symbiont density on total assimilated nitrogen (mg/recruit) was examined by plotting the marginal effect of symbiont density as moderated by symbiont ratio (S.m.:B.m.). Red bar indicates the range of symbiont ratios in which there is a significant negative effect of symbiont density on total assimialted nitrogen. Gray area shows 95% CI.

## Data Availability

All data and code are available at https://github.com/shelby26/NutrientDynamics.
