# Supplementary material for: Nutrient dynamics in coral symbiosis depend on both the relative and absolute abundance of Symbiodiniaceae species
Source: Microbiome. 2022 Nov 7;10:192. doi: 10.1186/s40168-022-01382-0 (PMC9639324; doi:10.1186/s40168-022-01382-0)
Supplement: Supplementary file 2 — Additional file 1: Table S1. Nutrient and isotopic compositions of artificial seawater media used for each stage of the experiment. Concentrations were based on Tanaka et al. (2006). All compounds were manufactured and purchased from Sigma-Aldrich ® (St. Louis, MO, USA). Fig. S1. Isotope baseline and dark controls. A set of recruits was tested to provide a baseline isotope value and to confirm that symbionts were the primer drivers of inorganic carbon and nitrogen into the symbiosis. Baseline recruits were sampled prior to the start of the experiment, Dark Enriched samples were exposed to isotopically enriched enriched nutriens but kept in the dark to inhibit photosynthesis; Enriched samples show the cumulative data from the final experiment. Fig. S2. The significant interaction effect of symbiont ratio and symbiont density on atom percent 13C values of symbiont tissues was examined by plotting the marginal effect of symbiont ratio (S.m.:B.m.) as moderated by symbiont density (cells/recruit). Green bar indicates the range of symbiont densities in which there is a significant positive effect of symbiont ratio on AP13Csym. Gray area shows the 95% CI. Fig. S3. The significant interaction effect of symbiont ratio and symbiont density on atom percent 15N values of host tissues was examined by plotting the marginal effect of symbiont density as moderated by symbiont ratio (S.m.:B.m.). Red bar indicates the range of symbiont ratios in which there is a significant negative effect of symbiont density on AP15Nhost. Gray area shows 95% CI. Fig. S4. The significant interaction effect of symbiont ratio and symbiont density on atom percent 15N values of symbiont tissues was examined by plotting the marginal effect of symbiont ratio (S.m.:B.m.) as moderated by symbiont density (cells/recruit). Red bar indicates the range of symbiont densities in which there is a significant negative effect of symbiont ratio on AP15Nsym. Gray area shows the 95% CI. Fig. S5. The significant interact [file 40168_2022_1382_MOESM1_ESM.pdf]

Table S1. Nutrient and isotopic compositions of artificial seawater media used for each stage of the experiment. Concentrations were based on Tanaka et al. (2006). All compounds were manufactured and purchased from Sigma-Aldrich ® (St. Louis, MO, USA).

**Stage**	**Concentration**	**Compound**
Acclimation<br>(unlabeled enriched)	N: $[NO_3]$=10µM<br>C: $[HCO_3]$=0.4mM	>99% $Na^{14}NO_3$<br>>99% $NaH^{12}CO_3$
Pulse<br>(labeled enriched)	N: $[NO_3]$= 9.07±0.8µM*<br>C: $[HCO_3]$=0.4mM	98% $Na^{15}NO_3$<br>98% $NaH^{13}CO_3$
Chase<br>(no nutrient addition)	N: $[NO_3]$= 1.59±0.27µM*<br>C: HCO_3 – not supplemented	--<br>--
*NO_3-N measured at Penn State Institutes of Energy & the Environment Water Quality Laboratory, University Park PA. Data are mean ± s.d. of experimental replicates.		

Supplemental Figures

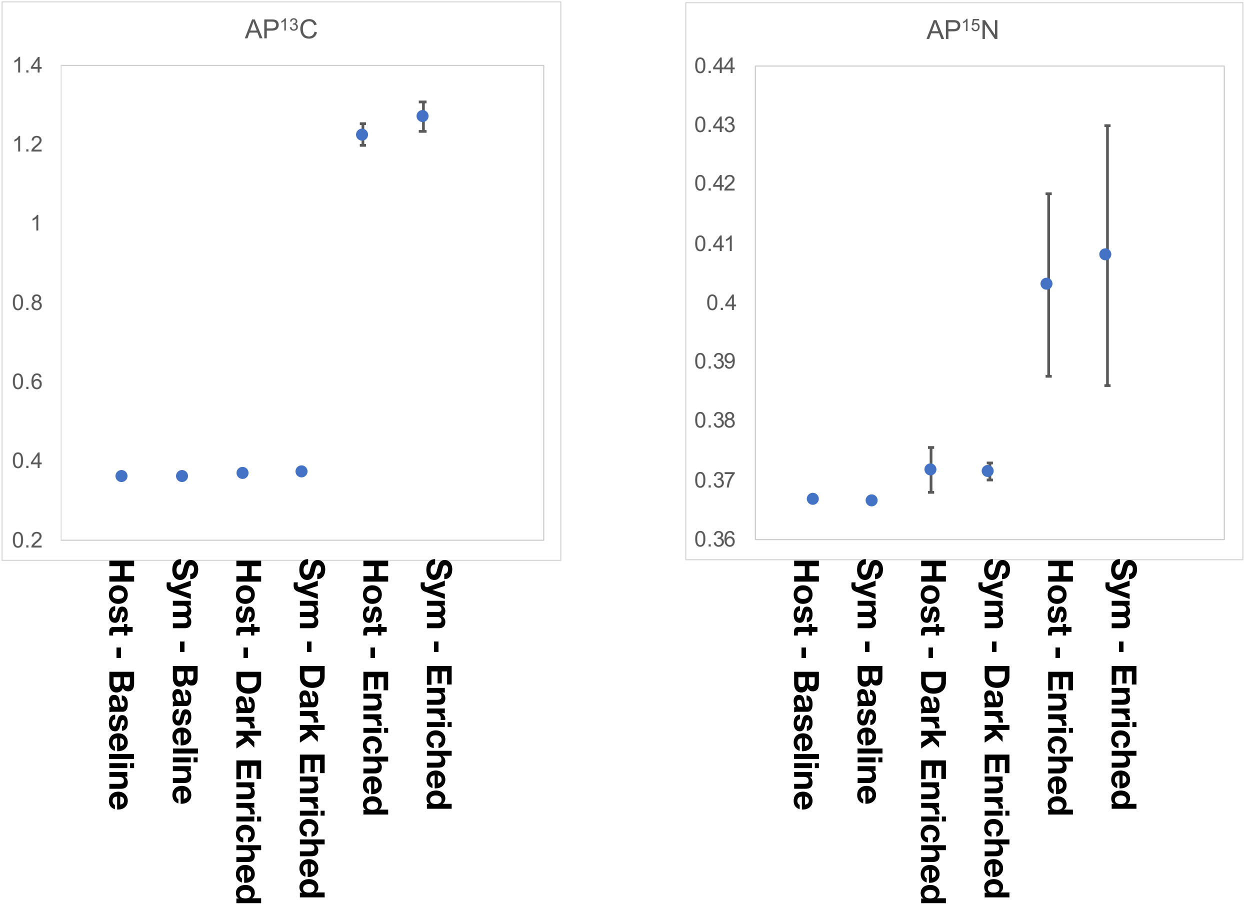

Fig. S1. Isotope baseline and dark controls. A set of recruits was tested to provide a baseline isotope value and to confirm that symbionts were the primer drivers of inorganic carbon and nitrogen into the symbiosis. Baseline recruits were sampled prior to the start of the experiment, Dark Enriched samples were exposed to isotopically enriched enriched nutriens but kept in the dark to inhibit photosynthesis; Enriched samples show the cumulative data from the final experiment.

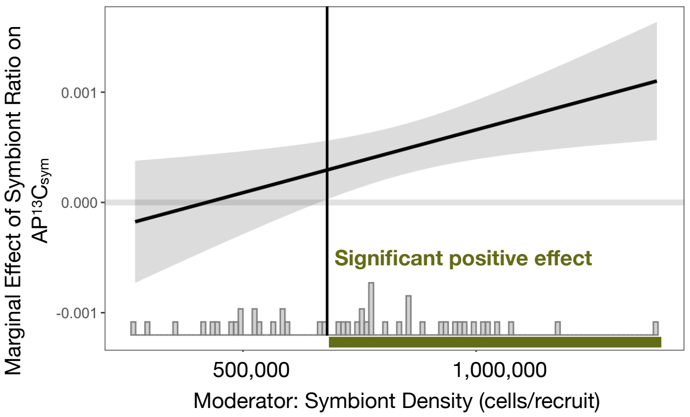

Fig. S2. The significant interaction effect of symbiont ratio and symbiont density on atom percent ^{13}C values of symbiont tissues was examined by plotting the marginal effect of symbiont ratio (*S.m.:B.m.)* as moderated by symbiont density (cells/recruit). Green bar indicates the range of symbiont densities in which there is a significant positive effect of symbiont ratio on $AP^{13}C_{sym}$. Gray area shows the 95% CI.

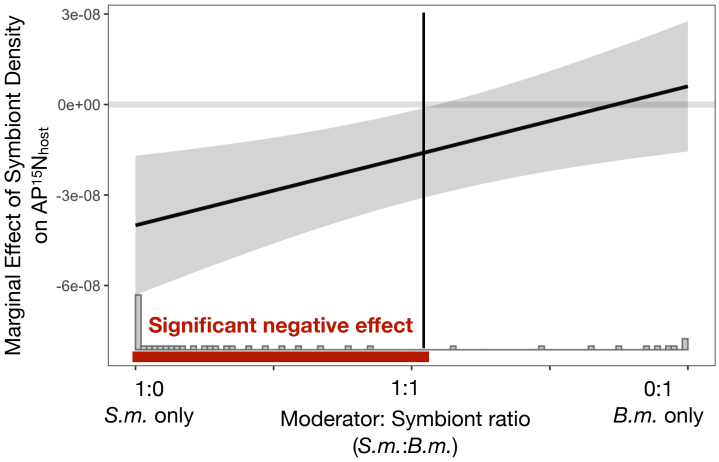

Fig. S3. The significant interaction effect of symbiont ratio and symbiont density on atom percent ^{15}N values of host tissues was examined by plotting the marginal effect of symbiont density as moderated by symbiont ratio (*S.m.:B.m.*). Red bar indicates the range of symbiont ratios in which there is a significant negative effect of symbiont density on $AP^{15}N_{host}$. Gray area shows 95% CI.

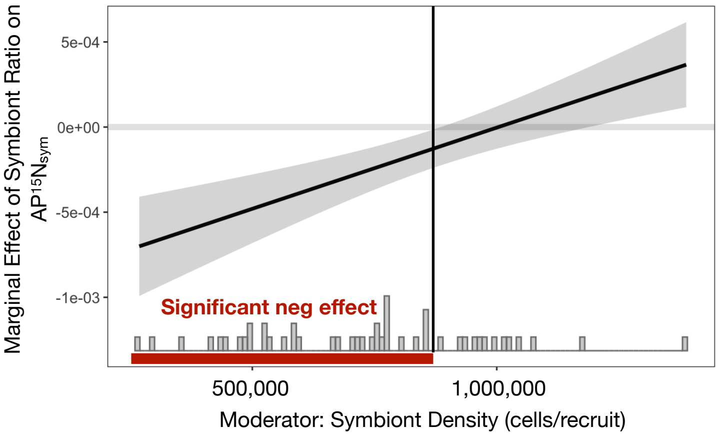

Fig. S4. The significant interaction effect of symbiont ratio and symbiont density on atom percent ^{15}N values of symbiont tissues was examined by plotting the marginal effect of symbiont ratio (*S.m.:B.m.)* as moderated by symbiont density (cells/recruit). Red bar indicates the range of symbiont densities in which there is a significant negative effect of symbiont ratio on $AP^{15}N_{sym}$. Gray area shows the 95% CI.

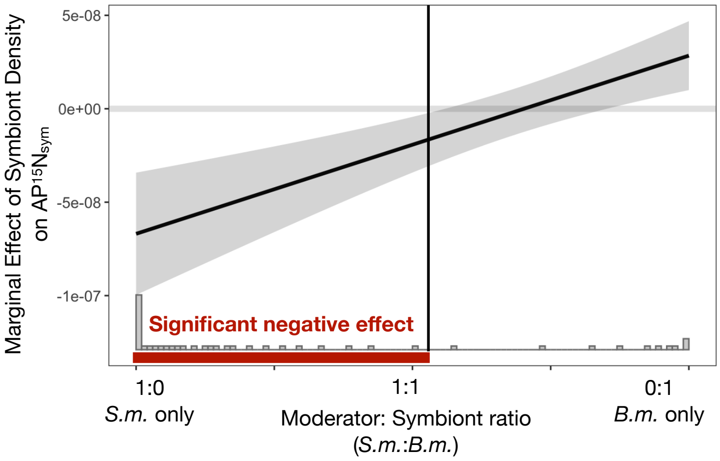

Fig. S5. The significant interaction effect of symbiont ratio and symbiont density on atom percent ^{15}N values of symbiont tissues was examined by plotting the marginal effect of symbiont density as moderated by symbiont ratio (*S.m.:B.m.*). Red bar indicates the range of symbiont ratios in which there is a significant negative effect of symbiont density on $AP^{15}N_{sym}$. Gray area shows 95% CI.

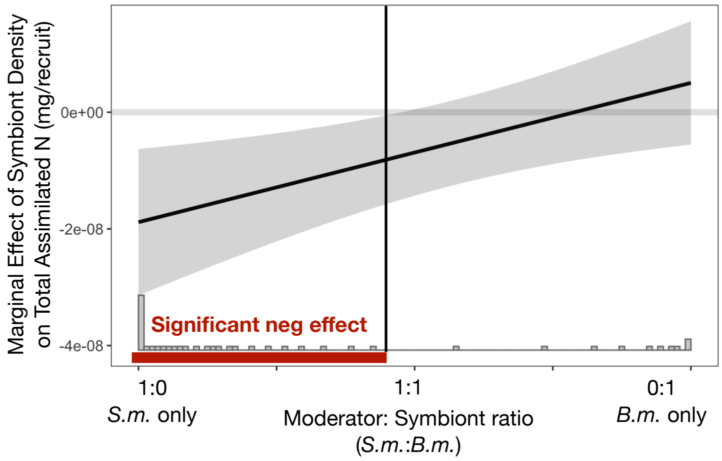

Fig. S6. The significant interaction effect of symbiont ratio and symbiont density on total assimilated nitrogen (mg/recruit) was examined by plotting the marginal effect of symbiont density as moderated by symbiont ratio (*S.m.:B.m.*). Red bar indicates the range of symbiont ratios in which there is a significant negative effect of symbiont density on total assimialted nitrogen. Gray area shows 95% CI.
